# Heterogenous effects of the Great East Japan earthquake on prosociality of people depending on their age

**DOI:** 10.1038/s41598-023-29536-6

**Published:** 2023-02-24

**Authors:** Yasuyuki Sawada, Toyo Ashida, Keiko Iwasaki

**Affiliations:** 1 Faculty of Economics, University of Tokyo, 7-3-1 Hongo, Bunkyo-Ku, Tokyo, 113-0033 Japan; 2 Hitotsubashi Institute for Advanced Study (HIAS), Hitotsubashi University, 2-1 Naka, Kunitachi, Tokyo 186-8601 Japan; 3 NLI Research Institute, 4-1-7 Kudankita, Chiyoda-Ku, Tokyo, 102-0073 Japan

**Keywords:** Psychology, Natural hazards

## Abstract

This study investigates the instability of prosociality in the real world by looking at the age-specific non-linear relationship between disaster exposure and prosocial behavior. We employed unique microdata from two communities in Japan that were hit by the Great East Japan Earthquake and Tsunami disaster in 2011. Exploiting exogenous variations in disaster exposure, we find age-specific heterogeneous effects of disaster exposure on prosocial behavior captured by the behavior of sending New Year’s cards as well as attitudinal survey questions. Among the older groups, disaster damages undermine prosociality, whereas the younger groups show reinforced prosocial behaviors. These findings can be explained consistently by combining two possible determinants of prosocial behavior: pure or impure altruism and self-enforcements in repeated interactions at workplaces. Age information can help disentangle these two elements at least partially.

## Introduction

Prosocial behavior plays a critical role in partially compensating for market and government failures^1–3^. It is therefore essential to measure the effects of large and extreme events, such as disasters and pandemics, on prosociality, especially when insurance and other market mechanisms function ineffectively. There is an growing body of literature that investigates the impact of substantial shocks such as economic crises, natural hazards, epidemics, and conflicts on people’s social preferences ^4^. However, the empirical findings are diverse and inconclusive^5^. Some studies find that a calamity makes people more prosocial^6–9^, whereas others indicate the opposite^10–12^. Andrabi and Das’s^13^ findings, however, were neutral. These findings suggest a non-linear relationship between shocks and social preferences. Reconciling the mixed evidence listed above^5^, Castillo and Carter^14^ claim that small shocks enhance prosocial behavior; however, greater shocks may decrease it.

This study attempts to bridge this gap in the literature by exploring the non-linear relationship between disaster and prosocial behavior from a unique perspective: an age gradient. Using survey questions to measure prosociality, Van Lange et al*.* ^15^ show that the prevalence of prosociality increased from early adulthood to middle adulthood and old age. Romano et al*.* ^16^ adopt laboratory experiments such as dictator and prisoner’s dilemma games to subjects of different ages, finding generally strong prosociality among the elderly. Mienaltowski and Wichman^17^ conduct iterated prisoner’s dilemma games with different age groups in anonymous or non-anonymous settings. They also show that older adults behave prosocially when compared with the young. However, Matsumoto et al*.* ^18^ explain that only a few studies have investigated the age gradient in determining prosocial behavior beyond early adulthood. A review by Lim and Yu^19^ reveals that there is a general lack of research into the nexus between prosociality and age, particularly in laboratory (lab) experiments with old-age subjects. They find that 94.7% of dictators in the meta-analysis were students and only 0.7% were elderly people. In addition, except for a related paper by Mienaltowski and Wichman^17^, there is almost no existing study that compares one-shot and repeated game settings of experiments played by the elderly. Reportedly, there has been no paper that explores the instability of prosociality in the real world across different age groups.

Our study aims to bridge this lacuna in the literature by exploiting natural experimental situations to identify the age-specific non-linear relationship between disaster exposure and prosocial behavior. Specifically, we analyze two communities in Japan utilizing exogenous variations in large-scale disaster damage to people’s homes, ranging from “*No significant damage*” to “*Totally collapsed.*” These are the city of Iwanuma, which encountered the Great East Japan Earthquake (GEJE) and Tsunami disaster of March 11, 2011, and the town of Futaba, which was impacted by both the GEJE and the resulting nuclear power plant failure. We employ unique data on real-world prosocial behavior captured by the action of sending New Year’s greetings. Carefully using age information among disaster victims, we believe that we can distinguish two types of behaviour. The first is prosociality based on altruism, and the second is prosocial behavior based on self-interested decisions.

There are two possible determinants behind the observed prosociality of sending New Year’s cards. The first, pure or impure altruism where impure altruism is a seemingly altruistic behavior driven by warm glow or self-image construction^7,20^ and second, self-enforcements in repeated interactions^21,22^. Theoretically speaking, disaster damage will undermine prosociality with (pure or impure) altruism because exposure to a disaster relatively decreases the marginal utility, i.e., the psychological benefit, from sending transfers^7^. In contrast, under the repeated game setting, we can see reinforced observed prosocial behavior with disaster exposure because the payoffs without cooperation are decreased directly due to disaster damage. This, in turn, makes self-enforcing cooperation more attractive, lowering the cooperation threshold of the subjective discount factor. i.e., the degree of patience, in a repeated game^21,22^.

The individuals’ ages can potentially play an important role in distinguishing these two cases. Among the older population with comparatively fewer remaining years, the repeated game mechanism is expected to be less important, and disaster exposure may reduce seemingly prosocial behavior further by increasing the probability of ending repeated interactions. However, self-enforcing cooperation can be sustained among the younger population^23^. Hence, disaster damage should undermine prosocial behavior uniformly for the older group, especially those who retired from their workplace and lost workplace social relationships. By contrast, among the younger group, who are in the process of building their social network through workplaces, the reinforced cooperation effects due to disaster exposure setting may dominate the immediate negative impact of damages on prosocial behavior, because of pure and impure altruism. Thus, we hypothesize that there is age-specific, or more directly, work-status-specific heterogeneity in prosocial behavior. As DellaVigna^24^ notes, it has been challenging to distinguish social preferences from repeated game strategies and other explanations in the field, and we believe that we make an important contribution.

Our findings relate to the age-specific heterogeneous effects of disaster exposure on prosocial behavior, which is evidenced by the behavior of sending New Year’s cards as well as attitudinal survey questions. Among the older groups, disaster damages undermine prosociality, whereas the younger groups show reinforced prosocial behaviors. These findings can be explained consistently by combining two possible determinants of prosocial behavior considered by us. While a substantial body of evidence on prosocial behavior has been accumulated through lab experiments, evidence from field research has been substantially limited. Little is known about mechanisms behind formation of prosociality^3,25,26^. Hence, we believe this study will contribute to the literature on prosocial behavior. A potential shortcoming of our analysis is the use of New year’s greetings cards as a measure of prosociality because it does not characterize prosociality toward strangers, which is typically measured in the literature. It instead measured the prosociality toward family or friends. To address this concern, we also employ alternative prosociality variables, that is, “generalized” or “universal” prosociality for further analyses.

This paper is organized as follows. Section "Data and Empirical Framework" describes the data and framework for empirical analysis, followed by the empirical results in Section "Results". Finally, section “Conclusions and remarks” summarizes concluding remarks.

## Data and empirical framework

To capture the level of real-world prosociality, we follow psychological studies on greeting card sending behavior^27^ and use data on the number of mailed New Year’s greeting cards. Clark’s^28^ study on the sociology of sympathy and other emotions, mentioned that “The underlying theme of all these cards, a theme explicitly stated in many, is that the sender is connected to the recipient, despite the temporary separation, figurative or literal, required by the plight. The fact that such cards are purchased and sent is evidence of sympathy-display norms.” Furthermore, studies in Japan discuss that New year’s cards form “closeness” networks^29,30^. Ishise and Sawada^31^ employ the number of letters posted to measure accumulated social capital through prosocial behavior. Accordingly, we believe that the New Year greetings cards’ variable could be a reasonable measure of “particularized prosociality.”

Although sending greetings cards on New Year is a custom in several countries, it has extraordinary significance in Japan; on average, a Japanese person sends about 25.2 New Year’s cards annually^32^. As New Year’s cards are expected to arrive by January 1, they need to be posted at least a week in advance. Sending cards timely entails the financial and non-financial costs of purchasing, writing, and sending them without immediate monetary benefits. Hence, the number of shipped cards can capture the level of prosociality. Moreover, sending greeting cards will not entail clear future benefits in general. We believe this provides a controlled setting to measure prosociality among a large population in the real world. Moreover, as the timely posting of cards requires commitment, the number of cards is partially affected by the degree of the present bias of each individual^33^.

### Two data sets

We employ our unique datasets from two disaster-affected communities in Japan. The first dataset is from Iwanuma, which was adversely affected by GEJE. It was obtained as part of a census run between November and December 2016 under the Japan Gerontological Evaluation Study (JAGES), a panel survey that started in 2010^33^. It enumerated all Iwanuma residents aged 65 years or older. Notably, World Health Organization defines people aged 65 years and above as the old population, and Japan’s official retirement age has been 65 years since 2013. Microdata in Iwanuma City was obtained as part of the JAGES^34–36^. The survey protocol was approved by the Human Subjects Committee of the Harvard T. H. Chan School of Public Health, the Ethics Committee of the Tohoku University Graduate School of Medicine, the Research Ethics Committee of the Graduate School of Medicine, Chiba University, and the Research Ethics Committee involving Human Participants of the Nihon Fukushi University. Respondents signed an informed consent form. We followed the STROBE Statement to report our observational study. Voluntary participation and the right to withdraw at any time were assured. This study conformed to the principles of the Declaration of Helsinki. We administered a postal questionnaire survey to all Iwanuma residents aged 65 years or older, between November and December 2016 (*N* = 7,421, response rate = 74.5%). As the 2016 data set does not contain home damage information, we acquired that data from the 2013 panel survey data (*N* = 2741). The sample size declined further because of missing observations in the dependent variables used in the analysis.

We collected the second dataset from Futaba in Fukushima Prefecture, which was located within 2–10 km radius of the Fukushima Daiichi Nuclear Power Plant. The GEJE impacted this community severely, as an evacuation order was placed immediately after the nuclear power plant failed as a consequence of the GEJE. With the support of the Futaba town office, in July 2016 we sent survey questionnaires to approximately 3,000 addresses listed as regular recipients of the town newsletter^33^. Recipients had full discretion regarding whether to return their (anonymous) completed questionnaires. Voluntary participation and the right to withdraw at any time were assured. This study conformed to the principles of the Declaration of Helsinki. In this scenario, informed consent was waived by the Ethics Committee of the Office for Life Science Research Ethics and Safety, the University of Tokyo. We followed the STROBE Statement to report our observational study.

### Empirical model

To test the hypothesis, we defined a treatment variable *d*, an ordered variable of the exposed disaster damage level that affects prosocial behavior in an age-specific manner. We set up the following analysis of covariance (ANCOVA) model to estimate the non-essential heterogeneous treatment effect:

1 $$Y_{it} = \alpha_{0} + a_{\delta }^{AGE} d_{i} + bY_{it - 1} + X_{it} \gamma \, + \, \varepsilon_{it} ,$$

where* Y*_*it*_ is a prosociality measure of individual *i* in year *t*, *d* is a variable representing exposure to a disaster, *Y*_*it-*1_ is a lagged proxy variable of the prosociality measure for individual *i* in year *t*-1, *X* is a set of observed control variables, and *ε* is a well-behaved error term. In Eq. (1), the disaster’s causal “treatment” effects on *Y* can be ascertained by the estimated parameter, *a*_*δ*_^*AGE*^, if disaster exposure *d* is orthogonal to the error term. Note that we allow heterogeneity of treatment effects depending on each respondent’s age or age group. If *a*_*δ*_^*AGE*^ > 0, disaster exposure aggravates an individual’s prosociality; if *a*_*δ*_^*AGE*^ < 0, it undermines prosociality. We allow this coefficient to be age specific to capture heterogeneous age effects. In addition, we explore that this heterogeneity depends on each respondent’s work status.

**Figure 1:**
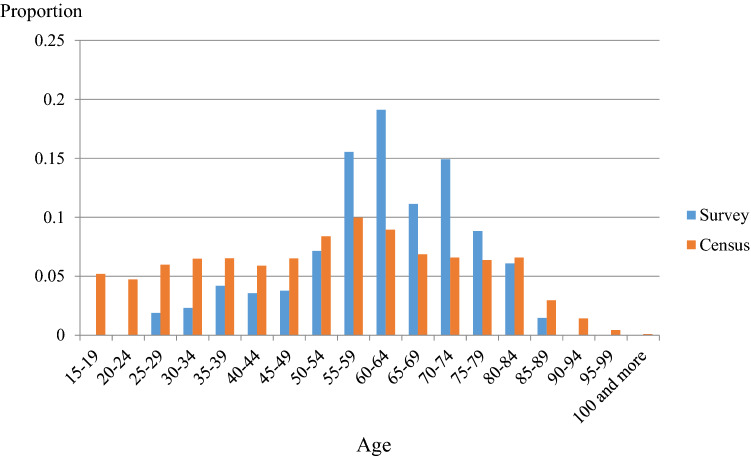
Age Distributions of Futaba Survey Respondents and Futaba Population Based on the 2010 Population Census of Japan. Data source: Futaba Survey Data and Japan’s 2010 Population Census.

When estimating Eq. (1), we need to consider sample selection bias arising from the Futaba data. According to the 2015 and 2020 Population Censuses, Futaba had approximately 6,900 residents and 2,600 households before the disaster in 2010, and approximately 6,600 residents and 2,300 households in 2015. The surveys were addressed only to the heads of households and 499 responses were received, with a 17% response rate. This response rate is not considered low relative to the general response rates for surveys in Japan. The actual response rate was higher than 17% since the 3,000 addresses included some duplications between heads of households and those who requested the newsletter. However, as seen in Fig. 1, the age distribution of respondents in our data was right skewed and concentrated around the median, compared to the actual population distribution of Futaba Town—based on Japan’s 2010 Population Census. To address possible sample selection bias in the data, we employed the control function approach with the standard Heckman correction term in our regression analyses, matching our data with the 2010 Census data for the datapoints of age, sex, and the residential areas in Futaba. The estimation procedure had two steps. In the first step, we included all the interactions in the three categorical variables as covariates for a survey response regression: age, sex, and the residential areas. In the second step, we estimated the main regression equation with the inverse Mills ratio, and adjusted the estimated variance and covariance matrix by the Bayesian parametric bootstrap method. The number of bootstrap replications was 400.

### Variables used in the analysis

The primary dependent variable in our analysis is the total number of 2016 New Year’s cards mailed (*Number of New Year’s cards mailed for the year 2016*). To measure this variable, we used each respondent’s answer for the following survey question in our surveys, “How many New Year’s cards for 2016 did you mail?” According to the Japan Post, the company sold 3.2 billion cards in 2016^32^, meaning that Japanese people sent around 30 New Year’s cards each. In our data, people sent an average of 37 and 29 cards, respectively, in Iwanuma and Futaba. This could reflect different age compositions (Table 1). According to the Japan Post^32^, people need to send New Year’s cards at least a week in advance (i.e., on or before December 25) because cards are ideally supposed to arrive on January 1. New Year’s cards mailed after December 15 are dealt separately from other outgoing mail. However, we also considered that people would not send New Year’s cards if they were mourning. To address this, as an independent variable, we included a dummy that takes the value of 1 if a respondent did not send out cards because they were grieving and 0 otherwise. This treatment is justifiable because mourning can be considered exogenous.

**Table 1:** Summary of descriptive statistics of iwanuma and futaba data.

Variable	Iwanuma					Futaba				
	Obs	Mean	Std. Dev	Min	Max	Obs	Mean	Std. Dev	Min	Max
*Y*_*it*_ (Number of New Year’s cards mailed)	2,368	37.27	54.45	0	830	388	28.80	38.64	0	300
Dummy = 1 if in mourning	2,368	0.11	0.36	0	1	388	0.13	0.34	0	1
*d* (Damage)	2,368	1.86	0.98	1	5	388	1.96	0.86	1	4
*Y*_*it-1*_ (General trust before disaster)	2,327	3.76	0.75	1	5	388	2.98	0.70	1	4
Homework	2,068	3.15	1.22	1	5	365	3.10	1.32	1	5
Volunteer	–	–	–	–	–	380	0.21	0.41	0	1
Dictator game	2,368	28.76	30.97	0	100	–	–	–	–	–
Work	2,368	0.10	0.31	0	1	384	0.29	0.46	0	1
Age	2,368	78.94	5.59	65	100	388	66.51	13.40	31	93

*Y*_*it*_ is the total number of New Year’s cards for 2016 that each respondent mailed. Higher values indicate more prosociality. *Y*_it-1_ indicates the pre-disaster level of social capital. Higher values signal more trust. Homework indicates the level of present bias when the participants were in junior high school, measured by the timing of doing their summer vacation homework. Higher values signal more present bias. *d* is the level of home damage caused by the disaster. A five-point scale is used to measure the Iwanuma data and a four-point scale to measure the Futaba data. Higher values represent more serious damage.

To gain further insights from empirical results using this New Year cards variable, we also employed the non-incentivized, hypothetical dictator game variable for the Iwanuma analysis, which is a response in percentage (0, 20, 40, 60, 80, or 100%) to a question: “*Suppose you receive a cash transfer of 5,000JPY from us. We would like to ask you to decide how to divide this money between you and someone (an anonymous person) randomly selected from your community. Out of 5,000 JPY endowments given to you, how much do you send to your anonymous partner? Identities of you and your partner are not revealed to each other.*” With a foreign exchange rate of 110 JPY per USD, 5,000 is approximately 45 USD. This question was included in the 2016 JAGES Survey in Iwanuma.

As the pre-disaster (lagged) dependent variable,* Y*_*it*-1_, of prosociality in estimating the ANCOVA model of Eq. (1), we include a general trust variable. It is based on the attitudinal survey questions on trust, following the General Social Survey (GSS) and the World Value Survey (WVS). It asked: “Generally speaking, would you say that most people in your community can be trusted?” and “generally speaking, would you say that most people can be trusted or that you can't be too careful in dealing with people?” in Iwanuma and Futaba, respectively. These are measured using 5-point and 4-point Likert scales, respectively, in Iwanuma and Futaba (Table 1). While we ask the GSS-WVS trust question in Futaba as a retrospective question on pre-disaster trust, our pre-disaster trust question in Iwanuma is about general trust toward “someone in your community” that was asked in the 2010 survey wave of the JAGES panel. Note that variants of these GSS-WVS trust questions have been widely used in the existing studies^13,37–41^.

As for the primary independent variable, damage level (*d*), our questionnaire requested the finding in the officially certified home-damage level report. The local government officially certifies each home’s damage level through detailed metric surveys designed by the central government. Hence, we believe that these damage level data are accurate even though they are self-reported. For the survey in Iwanuma, we provided five choices: (1) *No significant damage*, (2) *Partially damaged*, (3) *Half destroyed*, (4) *Nearly collapsed*, and (5) *Totally collapsed*. For the survey in Futaba, we provided four choices: (1) *No significant damage*, (2) *Partially damaged*, (3) *Half destroyed*, and (4) *Totally collapsed*. Based on the Futaba town office’s guidance, we merged the disaster damage categories “Nearly collapsed” with “Half destroyed,” following the damage level categories used for official reports on the GEJE by the Fire and Disaster Management Agency of the Ministry of Internal Affairs and Communications. We excluded those who did not answer the question about damage level from our analysis. We treated these damage variables as continuous.

Furthermore, considering that the subjective discount rate or impatience affects prosociality in the repeated games and that present bias can affect the number of New Year’s cards written and mailed before the deadline, we measured and included present bias or hyperbolic discounting before the disaster. We followed previous studies^42^ that use an individual’s timing for completing homework assignments during elementary and junior high school summer vacations (Sawada et al*.* ^33^; Supplementary Information, Figure S1). For the analysis, we treated our homework variable as continuous—the higher the value, the greater the level of present bias.

## Results

To clarify our identification strategy, we follow Callen^43^ to address two potential problems in identifying the causal impacts of disasters on prosociality. The first is a “selective exposure” problem where individuals may be located according to their preferences. To check the randomness of the treatment, *d*, we test the exogeneity of damage exposure by regressing the pre-disaster general trust variable against the level of home damage caused by the disaster (see Table 2). We can verify that there is no systematic correlation between the level of home damage and the baseline trust level, suggesting that the treatment, or disaster exposure, is randomly assigned. This is inconsistent with the existence of the selective exposure problem. Second, there may be a “selective migration” problem because individuals might selectively migrate out of affected zones based on their new preferences following the disaster. In Iwanuma data, almost no respondent migrated out and there is no attrition problem; therefore, issues of selective migration are not a major concern. In Futaba, by design, its mail-based survey does not have the selective migration problem. However, the relatively limited response level is a potential concern, and thus, we applied the control function approach to handle the sample selection problem.

**Table 2:** Baseline Balancing Test. Dependent variable: *Y*_t-1_ (General trust before the disaster).

Data	(1)	(2)	(3)	(4)
	Iwanuma	Iwanuma	Futaba	Futaba
*d* (damage)	0.0160	0.0160	0.00258	0.00258
	(0.0176)	(0.0194)	(0.0420)	(0.0387)
N	2,327	2,327	388	388
Adjusted R squared	0.000	0.000	− 0.003	− 0.003

Robust standard errors for columns 1 and 3, as well as cluster robust standard errors (clustered by 100 settled areas before the disaster in Iwanuma and 19 settled areas before the disaster in Futaba) for columns 2 and 4, are in parentheses.

* Significant at the 5% level ** Significant at the 1% level *** Significant at the 0.1% level.

The estimation results of Eq. (1) are summarized in Table 3.The Iwanuma data indicates that disaster exposure decreases the number of cards substantially—a one-unit increase in home damage leads to a 14.1% decline in the average number of cards [columns (1)]. In Futaba, although the relationship between disaster damage exposure and prosocial behavior is unclear for all age groups [columns (4)], we observe the same qualitative result as that in Iwanuma among those aged 65 and older [column (5)]. The disaster damage impact was more pronounced in Futaba, with a 29.6% decrease in the number of cards sent on average due to a one-unit increase in home damage. Intriguingly, among the group below 65 years of age, the relationship became positive and statistically significant [column (6)]. We verify that this difference may be driven by age-specific non-linearity since the interaction term of damage and age has a negative and marginally insignificant coefficient in specification [column (7)].

**Table 3:** Estimation results of regressing the number of New Year’s Cards on damage.

	(1)	(2)	(3)	(4)	(5)	(6)	(7)	(8)	(9)
Data:	Iwanuma	Iwanuma	Iwanuma	Futaba	Futaba	Futaba	Futaba	Futaba	Futaba
Dependent variable:	New Year’s Cards	New Year’s Cards	New Year’s Cards	New Year’s Cards	New Year’s Cards	New Year’s Cards	New Year’s Cards	New Year’s Cards	New Year’s Cards
Sample:	All (Age > = 65)	All (Age > = 65)	All (Age > = 65)	All	Age > = 65	Age < 65	All	Age > = 65	Age < 65
*d* (damage)	−5.279***	−4.852***	−6.247***	−2.923	−8.744***	6.945**	16.69	−11.22***	−1.322
	(1.291)	(1.298)	(1.642)	(2.721)	(2.300)	(3.451)	(13.69)	(2.919)	(4.391)
General trust before disaster	7.153***	6.233***	6.141***	9.829**	5.975**	16.03***	9.455**	6.041***	15.29***
	(1.761)	(1.735)	(1.708)	(3.688)	(2.779)	(5.760)	(3.736)	(2.017)	(4.514)
Homework	−1.189	−0.728	−0.590	−5.784*	−6.649**	−5.967	−5.731*	−4.644*	−6.424
	(1.569)	(1.586)	(1.574)	(3.130)	(2.752)	(4.563)	(3.067)	(2.682)	(4.686)
Age	−1.456***	−1.518***	−1.322***	0.143	0.0501	0.504*	0.686	0.259	0.913**
	(0.279)	(0.281)	(0.273)	(0.204)	(0.401)	(0.288)	(0.476)	(0.341)	(0.426)
Age × *d*		–	–				–0.291 +		
							(0.185)		
Volunteer	–	–	–					22.78***	3.091
								(4.798)	(6.258)
Dictator Game		0.347***	0.333***						
		(0.0706)	(0.0700)						
Work			0.251					14.54	–6.600
			(12.90)					(29.82)	(16.22)
Work × *d*			8.230					0.648	14.09**
			(5.384)					(14.80)	(6.432)
Inverse Mills Ratio		–	–	18.79	2.551	54.06**	19.87	7.587	57.40***
				(13.11)	(12.52)	(22.45)	(13.47)	(11.98)	(21.50)
N	2,327	2,327	2,327	6,047	6,047	6,047	6,047	6,047	6,047
N for second stage	–	–	–	388	234	154	388	234	154
Pseudo R squared	0.0405	0.0418	0.0430	0.0556	0.0551	0.0677	0.0562	0.0658	0.0746
Mean of the dependent variable	37.48	37.48	37.48	28.80	29.56	27.66	28.80	29.56	27.66

The dependent variable is the number of New Year’s cards mailed, which is considered a left-censored variable of prosociality. The dictator game variable is a response in percentage (0, 20, 40, 60, 80, or 100%) to a question, “Out of 5,000 JPY endowment, how much do you send to your anonymous partner.” Columns 1 to 3 present results using the Iwanuma data, and columns 4 to 9 display outcomes using the Futaba data. Cluster robust standard errors (clustered by 100 settled areas before the disaster in Iwanuma) are in parentheses for columns 1 to 3. Cluster bootstrap standard errors (clustered by 22 settled areas before the disaster in Futaba) are in parentheses for columns 4 to 9. The constant term is not presented. Other control variables are: a dummy variable for female, a missing dummy variable for sex, a dummy variable if a respondent was in mourning and did not mail out New Year’s cards, a dummy variable for missing data on homework, and a dummy variable to measure missing data of general trust before the disaster for all columns. Since we include the dummy to measure missing data, homework and volunteer include missing data, replaced by 0. Other control variables from columns 5 to 8 are house-type dummies. Those coefficients are not reported in the table but are available from the corresponding author upon request.

+ Significant at the 15% level * Significant at the 10% level, ** Significant at the 5% level, and *** Significant at the 1% level.

To explore the mechanisms behind the observed heterogeneous patterns of the damage and prosocial behavior nexus in the results based on the reduced-form model, we included additional control variables. First, a contemporaneous variable for altruism was added: sending amounts in the dictator game for Iwanuma and a binary variable that was 1 if a respondent engages in volunteer activities; and 0 otherwise in Futaba. These proxy variables for altruism had positive and significant coefficients for the elderly, suggesting that prosocial behavior could be explained by pure and impure altruism, at least partially [columns (2), (3), and (8)]. We believe that these specifications also showed the robustness of our results since our dependent variable and the lagged dependent variable are different. Inclusion of the altruism variables could mitigate remaining estimation biases arising from endogeneity and omitted variables.

Second, we included a dummy for work status and its interaction term with the damage variable [columns (3), (8), and (9)]. Presumably, for those with a repeated, long-term workplace relationship, we would see reinforced observed prosocial behavior with disaster exposure. This is because the decreased non-cooperation payoffs due to disaster damage made cooperation more attractive, lowering the cooperation threshold of the subjective discount factor, i.e., the degree of patience, in a repeated game. In these specifications, the interaction terms of the disaster damage variable and the work status dummy variable were all positive [columns (3), (8), and (9)] and statistically significant for the younger group below 65 years [column (9)]. This is consistent with our theoretical prediction. For the old age group, the relevant coefficients were not statistically significant, possibly due to the lack of sufficient variation in this variable [columns (3) and (8)]. In Iwanuma and Futaba, respectively, only 10% and 8.7% of the respondents continued to work.

We believe that our prosociality measure based on the number of cards, that is, “particularized prosociality,” does not characterize prosociality toward stranger or “generalized prosociality” or “universal trust,” but prosociality toward family or friends. This is a potential limitation of our contribution to the literature. To address this issue and to verify our interpretation of these findings further, Table 4 reports the estimation results using the contemporaneous GSS/WVS general trust variable and the sending amounts in an anonymous dictator game as the dependent variable. In contrast to particularized trust that can be determined within long-term individual relationships, generalized trust is more likely to be revealed in a one-shot game setting^44,45^. Therefore, we can hypothesize that the specifications with the general trust variable may exhibit a negative relationship between damage and prosociality regardless of age. Table 4 shows supportive evidence for this hypothesis in all specifications [columns (1), (2), (5), (6), (7), (8), (9), (10), (11), and (12)]. To gain further insights from Iwanuma data, we employed sending amounts in an anonymous dictator game as a dependent variable. The estimated results were comparable qualitatively [columns (3) and (4)]. As Yamagishi et al*.* ^46^ found strong consistency in Japanese people’s behaviors among different games including prisoner’s dilemma, trust, dictator, and faith games, we believe our findings suggest that the damage affects different aspects of prosociality simultaneously. With these “general prosociality” measure, we believe that the one-shot game mechanism could dominate the repeated game mechanism. The lack of significance on the estimated coefficient on the age-damage interaction variable and the across-the-board negative coefficients of the damage variable in Table 4 are consistent with the dominance of the one-shot game mechanism.

**Table 4:** Estimation results of regressing the GSS/WVS trust or the sending amount in the dictator game on damage.

	(1)	(2)	(3)	(4)	(5)	(6)	(7)	(8)	(9)	(10)	(11)	(12)
Data:	Iwanuma	Iwanuma	Iwanuma	Iwanuma	Futaba	Futaba	Futaba	Futaba	Futaba	Futaba	Futaba	Futaba
Dependent variable:	Trust	Trust	Dictator game	Dictator game	Trust	Trust	Trust	Trust	Trust	Trust	Trust	Trust
Sample:	All (Age > = 65)	All (Age > = 65)	All (Age > = 65)	All (Age > = 65)	All	Age > = 65	Age < 65	All	All	Age > = 65	Age < 65	All
*d* (damage)	−0.0330**	−0.0292*	−2.617***	−2.573***	−0.0954**	−0.0910*	−0.130**	−0.318	−0.0768**	−0.0751 +	−0.108**	−0.195
	(0.0152)	(0.0149)	(0.555)	(0.561)	(0.0415)	(0.0477)	(0.0583)	(0.225)	(0.0354)	(0.0496)	(0.0511)	(0.190)
General trust before disaster	0.423***	0.419***	3.974***	3.913***	0.359***	0.334***	0.380***	0.364***	0.354***	0.332***	0.373***	0.357***
	(0.0232)	(0.0232)	(0.921)	(0.915)	(0.0777)	(0.0868)	(0.135)	(0.0787)	(0.0787)	(0.0909)	(0.133)	(0.0800)
Homework	−0.0152	−0.0133	−1.202*	−1.176*	0.00427	−0.0611	0.0803*	0.00489	0.000539	−0.0629 +	0.0756 +	0.000949
	(0.0129)	(0.0126)	(0.626)	(0.626)	(0.0361)	(0.0434)	(0.0450)	(0.0360)	(0.0369)	(0.0388)	(0.0499)	(0.0370)
Age	0.000839	0.000925	0.258**	0.259**	−0.00258	−0.00201	0.00414	−0.00913	−0.00136	−0.00103	0.00474	−0.00485
	(0.00253)	(0.00248)	(0.115)	(0.116)	(0.00333)	(0.00879)	(0.00612)	(0.00644)	(0.00320)	(0.00875)	(0.00616)	(0.00565)
Age* d (damage)								0.00330				0.00175
								(0.00315)				(0.00270)
Income	–	0.000578***	−	0.00618	–	–	–	–	0.000889***	0.00182***	0.000583**	0.000871***
		(0.000117)		(0.00585)					(0.000337)	(0.000694)	(0.000248)	(0.000334)
Inverse Mills Ratio	–	–	–	–	0.214 +	0.170	0.272 +	0.199	0.156	0.135	0.219	0.149
					(0.146)	(0.240)	(0.188)	(0.151)	(0.142)	(0.259)	(0.190)	(0.147)
N	2,275	2,275	1,323	1,323	6,047	6,047	6,047	6,047	6,047	6,047	6,047	6047
N for second stage	–	–	–	–	418	255	163	418	418	255	163	418
Adjusted R squared	0.194	0.203	0.031	0.031	0.102	0.084	0.118	0.102	0.127	0.123	0.131	0.126

The dependent variable is the general trust. The dictator game variable is a response in percentage (0, 20, 40, 60, 80, or 100%) to a question, “Out of 5,000 JPY endowment, how much do you send to your anonymous partner.” Columns 1 to 4 present results using the Iwanuma data, and columns 5 to 10 display outcomes using the Futaba data. Cluster robust standard errors (clustered by 100 settled areas before the disaster in Iwanuma) are in parentheses for columns 1 to 4. Cluster bootstrap standard errors (clustered by 22 settled areas before the disaster in Futaba) are in parentheses for columns 5 to 10. The constant term is not presented. Other control variables are: a dummy variable for female, a missing dummy variable for sex, a dummy variable if a respondent was in mourning and did not mail out New Year’s cards, a dummy variable for missing data on homework, and a dummy variable to measure missing data of general trust before the disaster for all columns. Since we include the dummy to measure missing data, homework and volunteer include missing data, replaced by 0. Other control variables from columns (5) to (10) are house-type dummies. Those coefficients are not reported in the table but are available from the corresponding author upon request.

+ Significant at the 15% level * Significant at the 10% level, ** Significant at the 5% level, and *** Significant at the 1% level.

To quantify the magnitudes of the two mechanisms (i.e., altruism and repeated interaction) separately, we adopted Conti et al*.*’s^47^ mediation analysis framework by considering the case of a binary damage indicator. The contribution of a mediator, *x*, was computed using the formula ${\widehat{\gamma }}_{x}^{d=1}{\overline{x} }_{d=1}$-${\widehat{\gamma }}_{x}^{d=0}{\overline{x} }_{d=0}$, where $\widehat{\gamma }$_x_ is the estimated coefficient of *x* (allowing or not allowing heterogeneities depending on disaster exposure) and ${\overline{x} }_{d=1}$ and ${\overline{x} }_{d=0}$ are the subsample averages for *d* = 1 and *d* = 0, respectively, where *d* ≡ 1[“home collapsed” status], where 1[.] is an indicator function that takes 1 if the argument is true. Our results show that in the Iwanuma data, exposure to home damage would decrease the number of new year cards sent by 17.2 cards [Table 3, column (3)]. While deterioration of altruism captured by the change in the dictator game variable can explain 22.1% of this (a decrease of 3.8 cards), workplace participation reverses this by 19.3% (2.8 cards) on average [Table 3, column (3)]. Considering the homogenous age groups in Iwanuma, we compared extreme damage cases to detect the impacts clearly. For the mediation analysis in Futaba, we used estimated coefficients based on a binary home damage variable (Supplementary Inforamtion Table S1). In Futaba, home damage (*d* ≡ 1 [“home collapsed” or “half collapsed” damage status]) reduced the average number of cards sent by 14.1% among those above 65 years of age. Prosocial behavior through volunteer activities can explain 5.5% (-0.78 cards) of the decline on average, and the workplace effects reverse it by 2.0% (0.28 cards). Among those below 65 years of age in Futaba, disaster exposure increases the number of cards sent by 10.7%. Moreover, conditional on being employed, disaster exposure increases the number of cards by 14.1%. These results indicate altruism and work status as important mechanisms behind seemingly prosocial behaviors. These findings are broadly consistent with our theoretical hypothesis.

Furthermore, the coefficients for the homework variable were all negative in Table 3. The coefficient shows that those who are present biased tend to send fewer cards and are less prosocial, suggesting that present bias undermines prosocial behavior. As writing new year cards is a costly activity without immediate benefits, but with potential future rewards, it would be plausible to state that those who are present biased procrastinate on such activities.

## Concluding remarks

We find an age-specific heterogeneous effect of disaster exposure on prosocial behavior, which is consistent with combining two possible determinants of prosocial behavior: altruism and self-enforcements in repeated interactions. Moreover, the impact of disaster exposure is greater in Futaba than in Iwanuma, which may reflect differentiated exposure to disasters. Iwanuma residents were exposed only to the tsunami, whereas Futaba residents were affected by both the tsunami and displacement due to the nuclear power plant’s failure. As most areas in Futaba remain under a government-mandated evacuation order, residents had been prohibited from returning to their homes for over 5 years at the time of our surveys. In summary, we find a non-linear relationship between shocks and prosocial behavior, depending on age and the level of exposure. To externally validate our findings, it will be critical to examine the two mechanisms and the resulting non-linearities in the context of other disasters triggered by natural hazards, economic crises, and biological disasters including the ongoing COVID-19 pandemic in future studies^48^.

## Supplementary Information

Supplementary Information.

## Data Availability

All data needed to evaluate the conclusions in the study are described in the paper and/or the Supplementary Information. The JAGES data used in this study will be made available upon request, as per NIH data access policies. The authors require the applicant to submit an analysis proposal to be reviewed by an internal JAGES committee to avoid duplication. Confidentiality concerns prevent us from depositing our data in a public repository. Authors requesting access to the Iwanuma data need to contact the principal investigator of the parent cohort (Professor Katsunori Kondo) and the Iwanuma sub-study principal investigator (Professor Ichiro Kawachi) in writing (dataadmin.ml@jages.net). Proposals submitted by outside investigators will be discussed during the monthly investigators’ meeting to ensure that there is no overlap with ongoing analyses. If approval to access the data is granted, the JAGES researchers will request the investigator to financially compensate for our data manager's time to prepare the data for outside use. Data from Futaba Town is available upon request from Yasuyuki Sawada (sawada@e.u-tokyo.ac.jp) under a material transfer agreement with the University of Tokyo.
