# Supplementary material for: Heterogenous effects of the Great East Japan earthquake on prosociality of people depending on their age
Source: Sci Rep. 2023 Feb 24;13:3211. doi: 10.1038/s41598-023-29536-6 (PMC9950706; doi:10.1038/s41598-023-29536-6)
Supplement: Supplementary file 1 — Supplementary Information. [file 41598_2023_29536_MOESM1_ESM.pdf]

Supplementary Information for

Heterogenous Effects of the Great East Japan Earthquake on Prosociality of People Depending on Their Age

Yasuyuki Sawada*[a], Toyo Ashida[b], and Keiko Iwasaki[c]

Affiliations:

[a]*Corresponding author: Professor, Faculty of Economics, University of Tokyo, 7-3-1 Hongo, Bunkyo-ku, Tokyo 113-0033, Japan, E-mail: sawada@e.u-tokyo.ac.jp.

[b]Hitotsubashi University, 2-1 Naka, Kunitachi, Tokyo 186-8601, Japan, E-mail:t-ashida@umin.ac.jp

[c]NLI Research Institute, 4-1-7 Kudankita, Chiyoda-ku Tokyo 102-0073, Japan, E-mail: kiwasaki@nli-research.co.jp

This file includes:

Materials and Methods

Figures S1

Table S1

Materials and Methods

I. Measurement of Present Bias

II. Estimation Results Used for the Mediation Analysis for Futaba

I. Measurement of Present Bias

To measure the present bias or hyperbolic discounting before the disaster and the resulting procrastination behavior (Y_{t-1}), we followed previous studies[1–3] to capture each individual's timing for completing homework assignments during their elementary and junior high school summer vacations[4]. Elementary and junior high school education are compulsory in Japan. Since summer vacation is the longest holiday for students at that level, lasting approximately 40 days. Most schools provide a substantial amount of homework for students to do during the long vacation. Completing the homework depends on each student's self-motivation. We believe that, although it is not a pleasant task in most cases, it is the best measure to capture present bias or hyperbolic discounting during the respondents' adolescence. Specifically, we examined the response to the question, "*When did you work on your summer vacation homework when you were in elementary school?*" for respondents from Iwanuma. For participants from Futaba we asked, "*When did you work on your summer vacation homework when you were in junior high school?*" We asked them to choose from the following five choices: (1) *At the beginning of summer vacation*; (2) *Close to the beginning of summer vacation*; (3) *Equally every day*; (4) *Close to the end of summer vacation*; and (5) *At the end of summer vacation*. For the analysis, we treated the homework variable as a continuous variable where the higher the value, the greater the level of present bias. As we asked about respondents' attitudes regarding homework assignments retrospectively, measurement error could be a potential issue. With the possibility of the attenuation bias, we can still make reasonable inferences if the results are statistically significant.

To validate using the New Year's cards postmark date as a proxy for present bias, we examined the correlation between this variable and present bias measured by the Convex Time Budget (CTB) experiments developed by Andreoni and Sprenger[5]. The latter experimental data set was collected by incentivized artifactual field experiments, carried out in Iwanuma from February to May of 2017, with

a subset of the respondents taken from the Iwanuma census data used in this study[6–8]. A detailed description of the methodology can be found in Ashida et al.[6], Sawada and Kuroishi[7], and Kuroishi and Sawada[8]. We selected the subjects from a sample of individuals aged 65 years or older, who are cognitively and physically independent (that is, not certified as needing long-term care service). A total of 179 residents participated in our field experiments on February 8th (26 participants), 9th (11 participants), 10th (21 participants), 11th (16 participants), 14th (15 participants), 21st (26 participants), 27th (29 participants), and 28th (24 participants), as well as March 28th (6 participants) and 29th (5 participants) in 2017[8]. Figure S1 shows the relationship between the cards' postmark date on the horizontal axis and the quasi-hyperbolic discount factor, β, elicited by the CTB experiments on the vertical axis. We can see a negative relationship between these two variables; thus, it reasonably validates the variable of postmark date as a proxy measure of hyperbolic discounting.

References

[1] Ikeda, S., Kang, M. I. & Ohtake, F. Hyperbolic discounting, the sign effect, and the body mass index. *J. Health Econ.* **29**, 268–284; https://doi.org/10.1016/j.jhealeco.2010.01.002 (2010).

[2] Kang, M. I. & Ikeda, S. Time discounting and smoking behavior: Evidence from a panel survey(*). *Health Econ.* **23**, 1443–1464; https://doi.org/10.1002/hec.2998 (2014).

[3] Kang, M. I. & Ikeda, S. Time discounting, present biases, and health-related behaviors: Evidence from Japan. *Econ. Hum. Biol.* **21**, 122–136; https://doi.org/10.1016/j.ehb.2015.09.005 (2016).

[4] Sawada, Y., Iwasaki, K. & Ashida, T. Disasters aggravate present bias causing depression: Evidence from the Great East Japan Earthquake. *CIRJE Discussion Paper* F-1100. http://www.cirje.e.u-tokyo.ac.jp/research/dp/2018/2018cf1100.pdf (CIRJE, Tokyo, 2018) (Univ. of Tokyo, 2018).

[5] Andreoni, J. & Sprenger, C. Estimating time preferences from convex budgets. American. *Econ.*

Rev. **102**, 3333–3356; https://doi.org/10.1257/aer.102.7.3333 (2012).

[6] Ashida, T., Sawada, Y. & Kuroishi, Y. How does hyperbolic discounting affect human behavior? Evidence from the Great East Japan earthquake and tsunami using the convex time budget experiments. Paper presented at East Asian Economic Association's 15th Convention, November 5 and 6, 2016 (Bandung, Indonesia, 2016).

[7] Sawada, Y. & Kuroishi, Y. How to strengthen social capital in disaster affected communities? The case of the great East Japan earthquake in *Disaster Risks, Social Preferences, and Policy Effects: Field Experiments in Selected ASEAN and East Asian Countries, ERIA Research Project Report FY2013,* 34 (ed. Sawada, Y. & Oum, S.). https://www.eria.org/RPR_FY2013_No.34_Chapter_6.pdf 163–199 (2015).

[8] Kuroishi, Y. & Sawada, Y. On the stability of preferences: Experimental evidence from two disasters, *CIRJE Discussion Paper* F-1130. http://www.cirje.e.u-tokyo.ac.jp/research/dp/2019/2019cf1130.pdf (Faculty of Economics, the Univ. of Tokyo, 2019).

Figure S1: Relationship Between Present Bias Captured by Quasi-hyperbolic Discounting Factor,

Based on the Convex Time Budget Experiments and the Timing of Mailing New Year's Cards

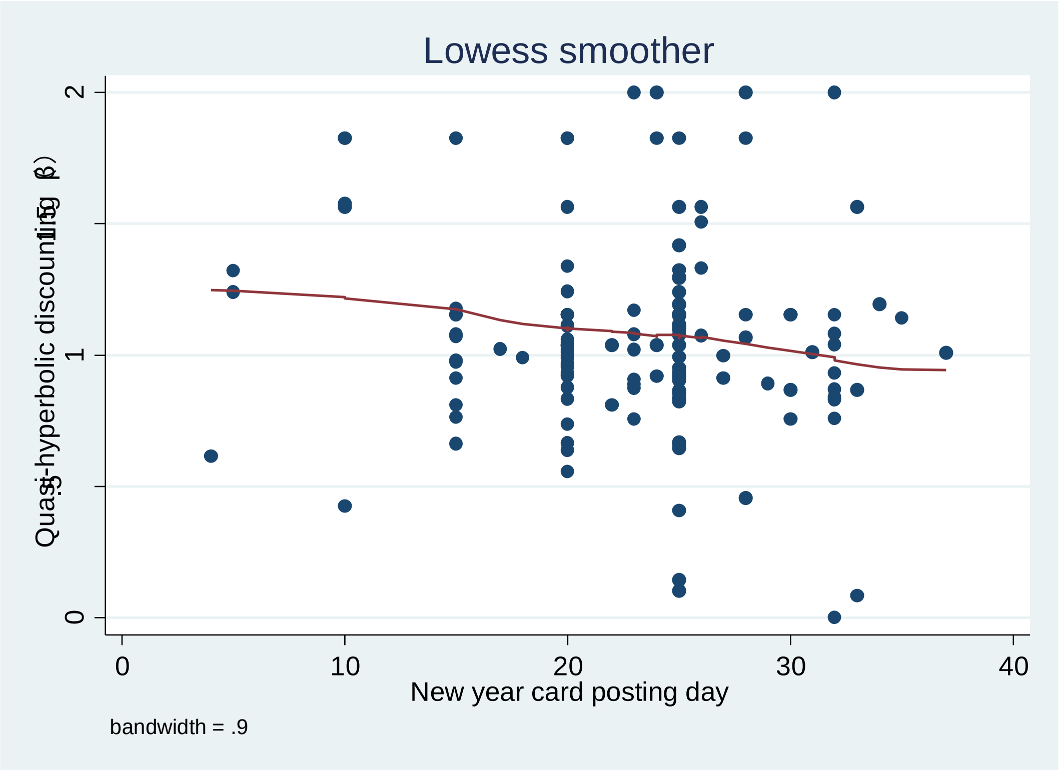

Note: The horizontal axis shows the day when New Year's cards were mailed for the first time and the vertical axis is the level of present bias captured by the quasi-hyperbolic discounting factor, β, based on the Convex Time Budget (CTB) experimental data taken from Sawada and Kuroishi[7] and Kuroishi and Sawada[8]. Following the Hadi's method, we identified and removed outliers, excluding two observations with more than 40 days.

II. Estimation Results Used for the Mediation Analysis for Futaba

Table S1. Estimation results of regressing the number of New Year's Cards on binary damage Variable

	(1)	(2)
Data:	Futaba	Futaba
Sample:	Age>=65	Age<65
Dummy=1 if there was no significant home damage/ the disaster caused partial home damage	Reference	Reference
Dummy=1 if the disaster destroyed half of the home/ the disaster destroyed the home	-19.18**	-1.658
	(7.464)	(11.28)
General trust before disaster	6.102***	14.07***
	(1.778)	(4.310)
Homework	-5.033*	-6.037
	(2.729)	(4.604)
Age	0.272	0.837**
	(0.323)	(0.412)
Volunteer	20.46***	-0.0726
	(5.392)	(5.096)
Work	16.46*	11.11
	(8.834)	(13.17)
Work × d	0.492	37.54**
	(18.09)	(17.43)
IMR	7.433	56.27***
	(10.97)	(19.63)
N	6,047	6,047
N for second stage	234	154
Pseudo R squared		
Mean of the dependent variable		

Notes: The dependent variable is the number of New Year's cards mailed, which is considered a left-censored variable of prosociality. Cluster bootstrap standard errors (clustered by 22 settled areas before the disaster in Futaba) are in parentheses. The constant term is not presented. Other control variables are: a dummy variable for female, a missing dummy variable for sex, a dummy variable if a respondent was in mourning and did not mail out New Year's cards, a dummy variable for missing data of homework and a dummy variable to measure missing data of general trust before the disaster, and house type dummies for all columns. Since we include the dummy to measure missing data, homework and volunteer include missing data, replaced by 0. Those coefficients are not reported in the table but are available from the corresponding author upon request.

+ Significant at the 15% level * Significant at the 10% level ** Significant at the 5% level *** Significant at the 1% level.
